# Exosomal lncRNA GAS5 regulates the apoptosis of macrophages and vascular endothelial cells in atherosclerosis

**DOI:** 10.1371/journal.pone.0185406

**Published:** 2017-09-25

**Authors:** Lei Chen, Wenjin Yang, Yijun Guo, Wei Chen, Ping Zheng, Jinsong Zeng, Wusong Tong

**Affiliations:** Department of Neurosurgery, The People's Hospital of Pudong New Area, Shanghai, PR China; Qatar University College of Health Sciences, QATAR

## Abstract

Atherosclerosis is universally recognized as a chronic lipid-induced inflammation of the vessel wall. Oxidized low density lipoprotein (oxLDL) drives the onset of atherogenesis involving macrophages and endothelial cells (ECs). Our earlier work showed that expression of long noncoding RNA-growth arrest-specific 5 (lncRNA GAS5) was significantly increased in the plaque of atherosclerosis collected from patients and animal models. In this study, we found that knockdown of lncRNA GAS5 reduced the apoptosis of THP-1 cells treated with oxLDL. On the contrary, overexpression of lncRNA GAS5 significantly elevated the apoptosis of THP-1 cells after oxLDL stimulation. The expressions of apoptotic factors including Caspases were changed with lncRNA GAS5 levels. Moreover, lncRNA GAS5 was found in THP-1 derived-exosomes after oxLDL stimulation. Exosomes derived from lncRNA GAS5-overexpressing THP-1 cells enhanced the apoptosis of vascular endothelial cells after taking up these exosomes. However, exosomes shed by lncRNA GAS5 knocked-down THP-1 cells inhibited the apoptosis of endothelial cells. These findings reveal the function of lncRNA GAS5 in atherogenesis which regulates the apoptosis of macrophages and endothelial cells via exosomes and suggest that suppressing the lncRNA GAS5 might be an effective way for the therapy of atherosclerosis.

## Introduction

Atherosclerosis, which can cause cardiovascular disease, is a leading cause of morbidity and mortality in industrialized country [1]. The onset of atherosclerosis is partly mediated by the dysfunction of endothelial cells (ECs) and infiltration of leukocytes, such as monocytes, which may differentiate into macrophages and dendritic cells. Meanwhile, the deposition of modified lipoproteins in the artery wall increases endothelial permeability and promotes the formation of foam cells and necrotic core along with lipid uptake of macrophages [2]. It is widely recognized that apoptosis in lesional macrophages, along with their defective function in efferocytosis, promotes plaque necrosis, which leads to plaque instability and thrombosis [3, 4]. Understanding the multifactorial process consisting of the interactions of several key components, including lipoproteins, inflammatory cells and vascular cells is extremely crucial [5].

Long noncoding (lnc) RNAs are generally defined as non-protein-coding RNAs that have at least 200 bp to 100 kb in length with highly conserved sequences [6, 7, 8]. LncRNAs have various functions including signaling transduction, molecular decoys, scaffolding and guiding ribonucleoprotein complexes. Accumulating evidences relate regulatory lncRNAs to human diseases [9, 10, 11]. A growing amount of studies have identified that lncRNAs regulate the functions of endothelial cells, macrophages, vascular inflammation and metabolism, suggesting the possibility of lncRNAs in influencing the progression of atherosclerosis [12]. The lncRNA growth-arrest specific transcript 5 (GAS5) is a 5′-terminal oligopyrimidine class of genes which regulates cell growth, proliferation and survival [13, 14]. The biogenesis of lncRNA GAS5 has been established. LncRNA GAS5 gene transcribes several snoRNAs as well as four splice variants of lncRNA GAS5 mRNA. However, due to the presence of STOP codon, none of the transcripts is transcribed into protein and degrade via the nonsense-mediated decay (NMD) pathway when translation is initiated. The RNA level of lncRNA GAS5 is regulated by its degradation instead of regulation at its transcriptional level [15]. One recent study identified that low expression of lncRNA GAS5 facilitated human saphenous vein smooth muscle cells proliferation and migration through Annexin A2 and thereby the pathogenesis of great saphenous veins varicosities [16].

Exosomes, small membrane particles (40–100 nm in diameter) originating from multivesicular bodies (MVBs), are generated from many cell types, and play key roles in the intercellular communication via the horizontal transfer of proteins, and RNAs to target cells [17]. Studies have discovered a list of statistically significant lncRNAs are differentially expressed in the exosomes in cancer [18]. Our earlier studies found that the molecular pathways underlying plaque formation in atherosclerosis were related to immune response, angiogenesis, cell proliferation, apoptosis and hypoxic microenvironments. And three lncRNAs, GAS5, SNHG6 and Zfas1, were significantly increased in the plaque of atherosclerotic patients [19]. LncRNA GAS5 has been found enriched in exosomes [20]. Here, the initial aim of this study was to investigate the function of lncRNA GAS5 in atherosclerosis progression. Whether macrophage derived exosomal lncRNA GAS5 modulate the function of endothelial cells in atherosclerosis is also investigated.

## Materials and methods

### Cell lines and cell culture

THP-1 cells and HUVEC were purchased from American Type Culture Collection (ATCC) and cultured in DMEM supplemented with 10% fetal bovine serum (Gibco, mexico). All the cells were cultured at 37°C in a humidified atmosphere with 5% CO2.

### Lentivirus mediated shRNA gene knockdown lncRNA GAS5

The stable knockdown lncRNA GAS5 cell lines were generated by transduction a lentiviral-mediated expression siRNA specific target of lncRNA GAS5. The targeted knockdown sequence was 5’-ggatgacttgcttgggtaa-3’. The virus transfected THP-1 cell lines with 8 μg/ml polybrene. After 48 hours, the cells are harvested, and the knockdown efficiency was tested by real-time-PCR.

### Lentivirus mediated over-expression of lncRNA GAS5

The stable over-expression of lncRNA GAS5 cell lines were generated by transduction a lentiviral-mediated overexpression lncRNA GAS5. The virus transfected THP-1 cell lines with 8μg/ml polybrene. After 48 hours, the cells are harvested, and the over-expression efficiency was tested by real-time- PCR.

### Real-time-PCR (RT-PCR)

Cellular RNA was isolated by Trizol-Reagent according to the manufacturer’s instructions. Briefly, the DNA was removed from the samples using DNase treatment (DNA-free kit; Ambion Applied Biosystems) and cDNA was synthesized from the purified RNA using Moloney murine leukemia virus reverse transcription kit (Promega). Gene-specific primer sets are listed in Table 1 and Actin primer sets are used to produce a normalization control. Real-time PCR was carried out in triplicate with the SYBR Green PCR Master Mix (Applied Biosystems) and a 7900HT Fast Real-Time PCR machine (Applied Biosystems).

**Table 1:** Primer sequences for RT-PCR.

GAS5-F	5’-ACACAGGCATTAGACAGAA-3’
GAS5-R	5’-CCAGGAGCAGAACCATTA-3’
Actin-F	5’-CACCATTGGCAATGAGCGGTTC-3’
Actin-R	5’-AGGTCTTTGCGGATGTCCACGT-3’

### Cell apoptosis assay

Apoptosis was determined by translocation of phosphatidylserine to the cell surface using an Annexin V-FITC/ Annexin V-PE and PI/7-ADD apoptosis detection kit (Nanjing KeyGen Biotech. Co. Ltd., China). The stable knockdown and over-expression lncRNA GAS5 THP-1 cells and its negative control cells were harvested and washed twice in cold PBS, and re-suspended in Annexin V-FITC/ Annexin V-PE and PI/7-ADD for 30 min in the dark. Cell apoptosis was analyzed by using Cell Quest software on a FACSAria Flow Cytometer (BD Inc., USA). Fluorescence was detected with an excitation wavelength of 480 nm.

### Western blot analysis

RIPA buffer in the presence of protease inhibitor cocktail and phosphorylation inhibitor cocktail were used to extract total protein. Appropriate mount protein was loaded into 10–15% SDS-polyacrylamide gel and transferred onto a nitrocellulose membrane (Millipore, Billerica, MA, USA). Primary antibodies were incubated overnight and secondary antibodies were incubated for 1 h at the appropriate dilutions. The signal was observed and developed with Kodak film by exposure to Enhanced Chemiluminescence (ECL) plus Western Blotting Detection Reagents (Amersham Biosciences, Piscataway, NJ, USA). Western blots were used to analyze the incorporation of each protein into cells. The antibodies against apoptosis associated Caspase3, Caspase7, Caspase9, P53 and actin used as control, were obtained from Cell Signaling Technology.

### Isolation and identification of exosomes released from the stable lncRNA GAS5 knocked-down or over-expressing THP-1 cells

80% confluent THP-1 cell lines cultured for an additional 48 hours in DMEM media deprived of FBS. The conditioned media of THP-1 cell lines was obtained and centrifuged at 300 × g for 10 min and 2000 × g for 10 min at 4°C to remove dead cells and cellular debris. Subsequently, the supernatant was filtered using a 0.45 μm filter sterilize Steritop TM (Millipore) to remove residual dead cells and cellular debris. Hereafter, the supernatant was centrifuged at 4000 × g at 4°C to about 200 μL by ultra-filtration in a 15 mL Amicon Ultra-15 Centrifugal Filter Unit (Millipore) and then centrifuged at 100,000 × g for one hour at 4°C to pellet the small vesicles. Exosomes were stored at − 80°C or used for downstream experiments. The protein concentration of the exosomes was determined using the Micro Bicinchoninic Acid (BCA) Protein Assay Kit (Thermo Fisher, Waltham, MA, USA).

### Effects of THP-1-exosomes on human vascular endothelial cells (HUVECs)

The exosomes (100 μg/mL) were labeled with PHK67 and incubated with HUVECs seeded onto 24-well plates. After 24 hours (t = 24 hours), the HUVECs were then photographed under a fluorescence microscope (Leica AF6000). The results were analyzed by observing the fluorescence into the cells.

Influence of exosomes on the expression of lncRNAGAS5 genes of HUVECs was evaluated with RT-PCR. And influence of exosomes on apoptosis of HUVECs was analyzed by using the Flow Cytometer.

### Statistical analysis

For quantitative data, all results were expressed as the mean SD. Statistical significance between groups was determined using the Student’s t-test using SPSS 18.0 (SPSS, USA). Each experiment was repeated at least three times. P < 0.05 was considered statistically significant.

## Results and discussion

### Knockdown of lncRNA GAS5 reduced the apoptosis of THP-1 cells treated with oxLDL

**Fig 1:**
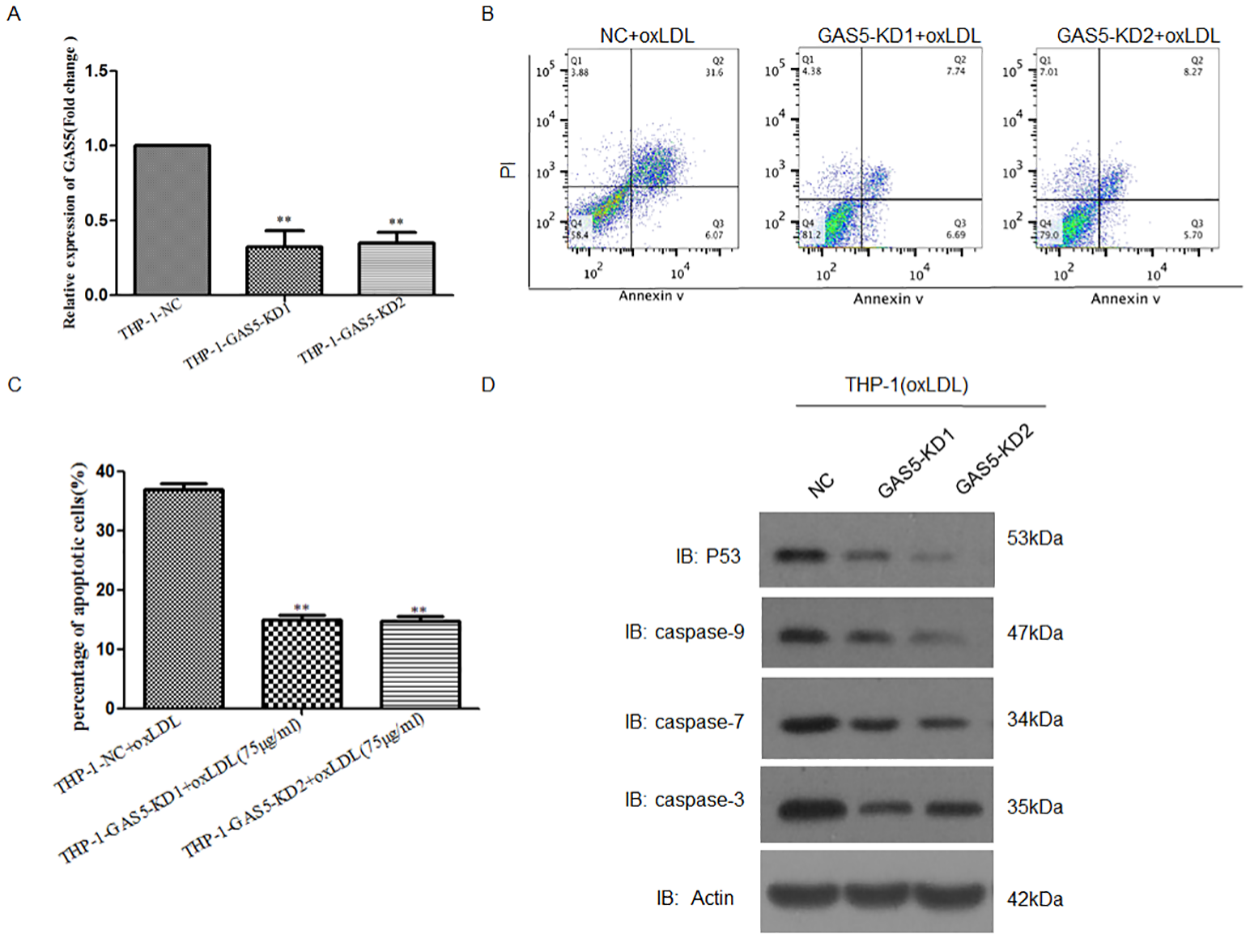
LncRNA GAS5 knocked-down THP-1 cells treated with 75 μg/mL oxLDL reduced cell apoptosis. **A**. RT-PCR analysis of lncRNA GAS5 expression in NC and lncRNA GAS knocked-down cells. More than 70% of lncRNA GAS5 expression was decreased in knocked down cells. **P < 0.01, THP-1-GAS5-KD vs. NC Control. **B** and **C**. Flow cytometry analysis of the percentage of apoptotic THP-1 cells with lncRNA GAS5 knocked-down after treated with oxLDL (75μg/mL). **P < 0.01, ***P < 0.001, THP-1-GAS5-KD vs. NC control. **D**. Western blot analysis of P53, Caspase 3, Caspase 7 and Caspase 9 in NC and lncRNA GAS5 knocked-down cells treated with oxLDL (75μg/mL).

Using lentivirus mediated shRNA gene knockdown system, we knocked down lncRNA GAS5 expression in THP-1 cells. From the mRNA level, more than 70% of lncRNA GAS5 expression was decreased in gene knockdown system (Fig 1A). Then, we treated lncRNA GAS5 knocked-down THP-1 cells with 75μg/mL oxLDL (Intracel Resources, Frederick, MD, USA) for 24 h and detected the percentage of apoptotic cells using flow cytometry. Compared with its negative control cells, we found that the percentage of apoptotic lncRNA GAS5 knocked-down THP-1 cells was increased after treated with oxLDL (Fig 1B and 1C). Western blot results demonstrated that the expressions of P53, Caspase 3, Caspase 7 and Caspase 9 were reduced after inhibition of lncRNA GAS5 expression in response to oxLDL stimulation (Fig 1D).

### Over-expression of lncRNA GAS5 increased the apoptosis of THP-1 cells treated with oxLDL

**Fig 2:**
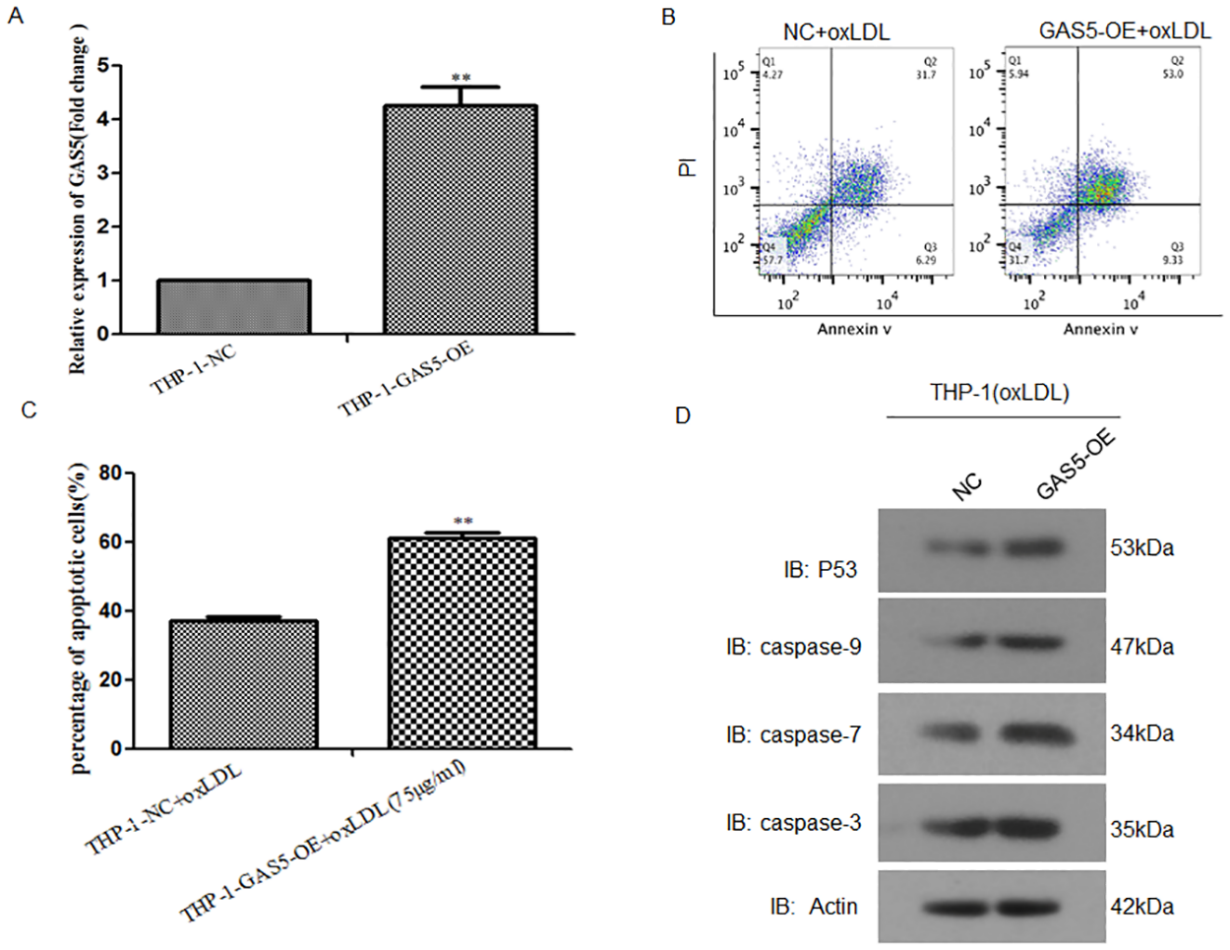
LncRNA GAS5 over-expressing THP-1 cells treated with 75 μg/mL oxLDL elevated cell apoptosis. **A**. RT-PCR analysis of lncRNA GAS5 expression in NC and lncRNA GAS5 overexpressing cells. More than four folds of lncRNA GAS5 expression was increased in over-expressing cells. **P < 0.01, THP-1-GAS5-OE vs. NC control. **B** and **C**. Flow cytometry analysis of apoptotic THP-1 cells with lncRNA GAS5 over-expressing after treated with oxLDL (75μg/mL). **P < 0.01, THP-1-GAS5-OE vs. NC control. **D**. Western blot analysis of P53, Caspase 3, Caspase 7 and Caspase 9 in NC and llncRNA GAS5 overexpressing cells treated with oxLDL (75μg/mL).

Meanwhile we over expressed lncRNA GAS5 expression in THP-1 cells using lentivirus mediated over-expression system. At the mRNA level, lncRNA GAS5 expression was increased more than 4 folds in gene over-expression system (Fig 2A). Then, we treated lncRNA GAS5 over-expressing THP-1 cells with 75μg/mL oxLDL for 24 h and detected the percentage of apoptotic cells using flow cytometry. Compared with its negative control cells, we found that after treated with oxLDL, the percentage of apoptotic lncRNA GAS5 over-expressing THP-1 cells was increased (Fig 2B and 2C). Western blot results showed that the expressions of P53, Caspase 3, Caspase 7 and Caspase 9 were upregulated after lncRNA GAS5 over-expressing (Fig 2D). These results highlighted the importance of lncRNA GAS5 in the regulation of macrophage apoptosis and suggested that lncRNA GAS5 may have implications for the therapy of atherosclerosis.

### Identification of lncRNA GAS5 in THP-1 derived exosomes treated with oxLDL

**Fig 3:**
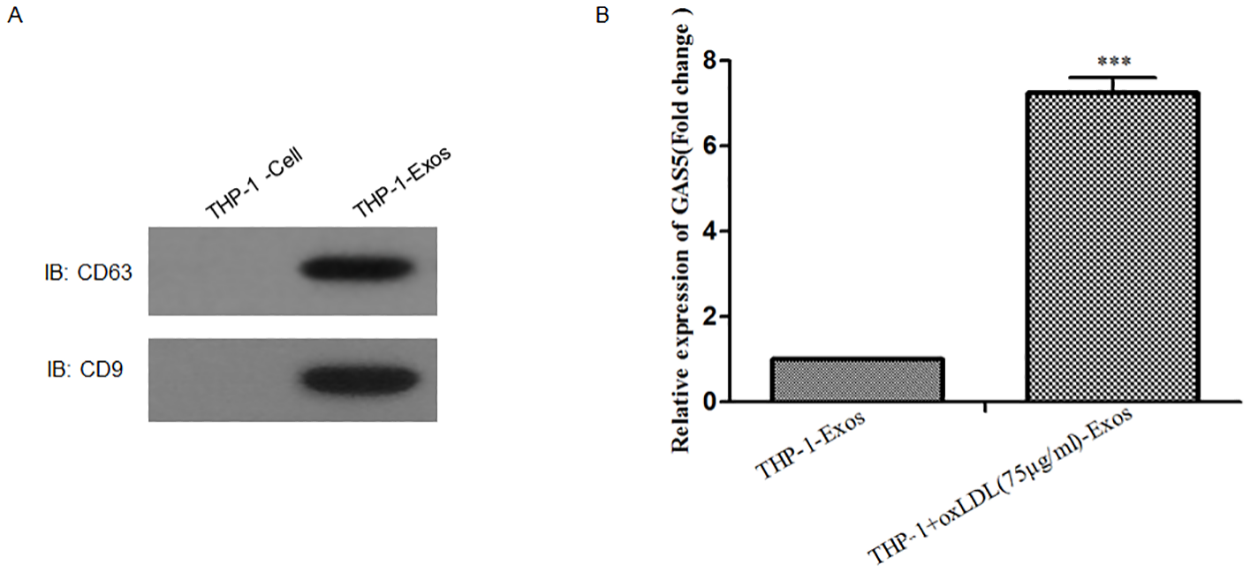
Identification of THP-1-Exos and expression of lncRNA GAS5 in exosomes derived from oxLDL treated THP-1 cells. **A**. Western blotting analysis of exosomal surface marker proteins CD63 and CD9 in THP-1-Exos. **B**. RT-PCR analysis of lncRNA GAS5 gene expression profoundly up-regulated in the THP-1-Exos after oxLDL (75μg/mL) stimulation 24h. **P < 0.001, THP-1+oxLDL-Exos vs. NC control.

Western blotting analysis showed that exosomes isolated from the THP-1 cultured medium expressed characteristic exosomal surface marker proteins: CD63 and CD9 (Fig 3A). To examine whether lncRNA GAS5 exist in THP-1 cells derived exosomes and whether the levels of lncRNA GAS5 in these exosomes altered by oxLDL treatment, RT-PCR analysis was performed. To our surprise, lncRNA GAS5 was found in THP-1 derived exosomes. More, compared to the control group, lncRNA GAS5 was profoundly up-regulated after oxLDL treatment (Fig 3B). These results indicate that lncRNA GAS5 was markedly enriched in the THP-1-exosomes after oxLDL stimulation.

### Exosomes derived from lncRNA GAS5 knocked down THP-1 cells reduced the apoptosis of vascular endothelial cells

**Fig 4:**
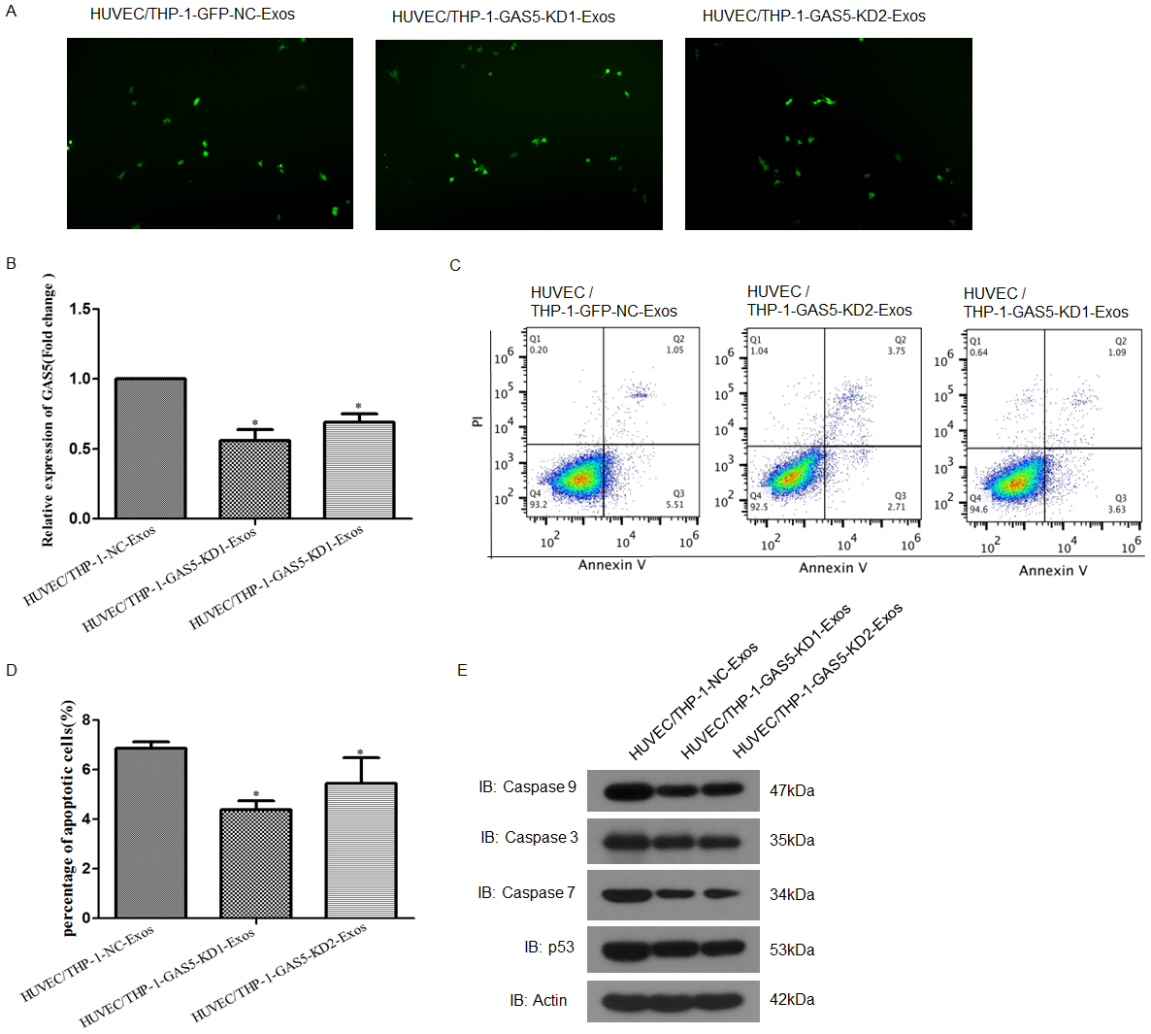
HUVECs incubated with exosomes (100 μg/mL) derive from lncRNA GAS5 knocked down THP-1 cells reduced cell apoptosis. **A**. Fluorescence microscopy analysis showed the GFP-labeled THP-1-exosomes were taken up and transferred to the perinuclear region of endothelial Cells. The magnification was 100 x. **B**. RT-PCR analysis of lncRNA GAS5 expression in HUVEC cells incubated with different exosomes. HUVEC cells incubated with exosomes derived from the lncRNA GAS5 knocked-down THP-1 cells contained markedly reduced lncRNA GAS5 levels. *P < 0.05, HUVEC-GAS5-KD vs. NC control. **C** and **D**. Flow cytometry analysis of HUVEC cells incubated with the exosomes derived from the knockdown lncRNA GAS5 THP-1 reduced the percentage of apoptotic cells. *P < 0.05, HUVEC-GAS5-KD vs. NC control. **E**. Western blot analysis of P53, Caspase 3, Caspase 7 and Caspase 9 in cells treatment with the exosomes derived from lncRNA GAS5 knocked-down THP-1.

Furthermore, fluorescence microscopy analysis showed the GFP-labeled THP-1-exosomes were taken up and transferred to the perinuclear region of vascular endothelial cells (Fig 4A), indicating that the THP-1-exosomes might influence the function of vascular endothelial cells. The expression of lncRNA GAS5 in vascular endothelial cells was determined by RT-PCR analysis, and the results showed that compared with control, endothelial cells incubated with exosomes isolated from the cultured medium of lncRNA GAS5 knocked down THP-1 cells contained lower lncRNA GAS5 (Fig 4B). Cell apoptotic assay was used to examine the effect of THP-1-exosomes with different levels of lncRNA GAS5 on the apoptosis of endothelial cells. The results showed that the HUVEC cells incubated with exosomes isolated from the lncRNA GAS5 knocked down THP-1 cells cultured medium reduced the percentage of apoptotic HUVECs compared with wild-type control (Fig 4C and 4D). To further analyze whether apoptosis-related genes were altered, Western blot analysis was performed. The expressions of P53, Caspase 3, Caspase 7 and Caspase 9 were profoundly down-regulated after treated with the exosomes isolated from the lncRNA GAS5 knocked down THP-1 cells compared with negative control (Fig 4E).

### Exosomes derived from lncRNA GAS5 overexpressing THP-1 cells elevated the apoptosis of vascular endothelial cells

**Fig 5:**
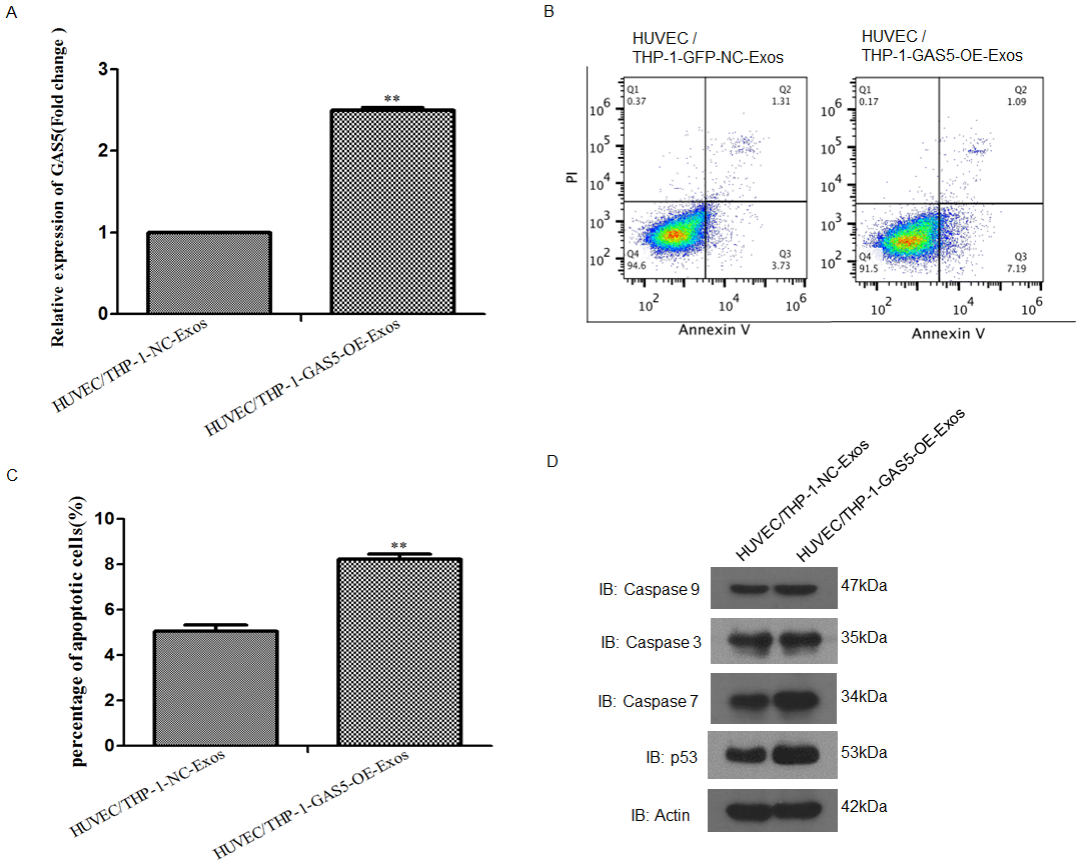
HUVECs incubated with exosomes (100 μg/mL) derive from the over-expressing lncRNA GAS5 THP-1 enhanced cell apoptosis. **A**. RT-PCR analysis of lncRNA GAS5 expression of HUVECs incubated with exosomes derived from NC or lncRNA GAS5 overexpressing THP-1 cells. HUVEC cells incubated with exosomes derived from the lncRNA GAS5 overexpressing THP-1 cells contained markedly increased lncRNA GAS5 levels. *P < 0.01, HUVEC-GAS5-OE vs. control. **B** and **C**. Flow cytometry analysis of HUVEC cells incubated with the exosomes derive from the over-expressing lncRNA GAS5 THP-1 cells enhanced the percentage of apoptotic cells. **P < 0.01, HUVEC-GAS5-OE vs. control. **D**. Western blot analysis of P53, Caspase 3, Caspase 7 and Caspase 9 in cells treatment with the exosomes derived from the lncRNA GAS5 overexpressing THP-1 cells.

The expression of lncRNA GAS5 in vascular endothelial cells was determined by RT-PCR analysis, the results showed that exosomes isolated from the over-expressing lncRNA GAS5 THP-1 cells markedly increased the lncRNA GAS5 expression in HUVECs compared to control (Fig 5A). Cell apoptotic assay was used to examine the effect of these exosomes on the apoptosis of endothelial cells, the results found that the exosomes isolated from the lncRNA GAS5 over-expressing THP-1 cells enhanced the percentage of apoptotic HUVECs (Fig 5B and 5C). To further analyze whether apoptosis-related genes expression was altered in HUVEC cells after internalization of these exosomes, Western blot analysis was performed. The expressions of P53, Caspase 3, Caspase 7 and Caspase 9 were up-regulated after treated with the exosomes isolated from the lncRNA GAS5 over-expressing THP-1 cells (Fig 5D). These results indicated that lncRNA GAS5 exist in THP-1 cells derived exosomes and exosomal lncRNA GAS5 modulated the apoptosis of endothelial cells.

Atherosclerotic process is related to pro-atherogenic and pro-inflammatory mediators that lead to formation of plaques and progressive stenosis [21, 22]. Macrophages are involved in all stages of plaque development [23]. The initial step of atherosclerosis involves high levels of low density lipoprotein (LDL) which are oxidized and recruit monocytes. Oxidized LDL (oxLDL) induces expression of adhesion molecules on endothelium and facilitates monocyte adhesion to intima, determining the extension of lesion formation and progression [24, 25]. One recent study identified that low expression of the newly found lncRNA GAS5 can promote the proliferation and migration of human saphenous vein smooth muscle cells [16]. Our earlier work showed that lncRNA GAS5 also play a role in the progression of atherosclerosis [19]. In the present study, we found that lncRNA GAS5 regulates the apoptosis of oxLDL-treated THP-1 cells. In these cells, high level of lncRNA GAS5 expression promotes basal apoptosis, whereas low level of lncRNA GAS5 inhibits apoptosis. The following study further extends these findings by demonstrating that the vascular endothelial cells apoptosis are quantitatively related to THP-1 derived exosomal lncRNA GAS5 levels, suggesting that exosomes may act as a ‘master regulator’ of lncRNA GAS5.

The monolayer of endothelial cells (ECs), lining in the innermost part of the arterial vessel, play a pivotal role in maintaining the homeostasis of vessel when provoked with many stimuli, such as oxidized LDLs, inflammation and shear stress [26]. The activation and dysfunction of ECs triggered by those stimuli indicate the initiation of atherosclerosis progress. A recent study showed that lncRNAs may influence the endothelial function and ECs senescence, which is a key character of atherosclerosis plaque [27]. Herein, we revealed that THP-1 derived exosomal lncRNA GAS5 transplantation could enhance the apoptosis of vascular endothelial cells while low lncRNA GAS5 transplantation could inhibit the apoptosis of vascular endothelial cells. Therefore, our findings highlighted the importance of exosomal pathway for macrophages and vascular endothelial cells communicating with each other by mediating lncRNA GAS5 in atherosclerosis.

## Conclusions

As membrane vesicles, exosomes are crucial in the intercellular communications and may be a key mediator of lncRNA GAS5, which provide the possibility of an alternative strategy for treatment of atherosclerosis.

## References

[1] Rafieian-Kopaei M, Setorki M, Doudi M, Baradaran A, Nasri H. Atherosclerosis: process, indicators, risk factors and new hopes. Int. J. Prev. Med. 2014;5:927e946. 25489440 PMC4258672

[2] Novak J, Bienertova-Vasku J, Kara T, Novak M. MicroRNAs involved in the lipid metabolism and their possible implications for atherosclerosis development and treatment. Mediat. Inflamm. 2014; 275867. 10.1155/2014/275867 PMC4020222 24876669

[3] Toba H, Cortez D, Lindsey ML, Chilton RJ. Applications of miRNA technology for atherosclerosis. Curr. Atheroscler. Rep. 2014;16:386 doi: 10.1007/s11883-013-0386-9 24395388 10.1007/s11883-013-0386-9 PMC4362664

[4] Back M, Hansson GK. Anti-inflammatory therapies for atherosclerosis. Nat. Rev. Cardiol. 2015;12:199e211. 25666404 10.1038/nrcardio.2015.5

[5] Aryal B, Rotllan N, Fernandez-Hernando C. Noncoding RNAs and atherosclerosis. Curr. Atheroscler. Rep. 2014;16:407 doi: 10.1007/s11883-014-0407-3 24623179 10.1007/s11883-014-0407-3 PMC4145585

[6] Lander ES, Linton LM, Birren B, Nusbaum C, Zody MC, Baldwin J, et al Initial sequencing and analysis of the human genome. Nature. 2001;409 (6822):860–921. doi: 10.1038/35057062 11237011 10.1038/35057062

[7] Mattick JS. The functional genomics of noncoding RNA. Science. 2005;309:1527–1528. doi: 10.1126/science.1117806 16141063 10.1126/science.1117806

[8] Ma H, Hao Y, Dong X, Gong Q, Chen J, Zhang J, et al Molecular mechanisms and function prediction of long noncoding RNA. Scientific World Journal. 2012;541786 doi: 10.1100/2012/541786 23319885 10.1100/2012/541786 PMC3540756

[9] Chen X, Yan GY. Novel human lncRNA-disease association inference based on lncRNA expression profiles. Bioinformatics. 2013;29 (20): 2617–2624. doi: 10.1093/bioinformatics/btt426 24002109 10.1093/bioinformatics/btt426

[10] Arase M, Horiguchi K, Ehata S, Morikawa M, Tsutsumi S, Aburatani H, et al Transforming growth factor-beta-induced lncRNA-Smad7 inhibits apoptosis of mouse breast cancer JygMC(A) cells. Cancer Sci. 2014; 105 (8):974–982. doi: 10.1111/cas.12454 24863656 10.1111/cas.12454 PMC4317863

[11] Liu Q, Huang J, Zhou N, Zhang Z, Zhang A, Lu Z, et al LncRNA loc285194 is a p53-regulated tumor suppressor. Nucleic Acids Res. 2013;41 (9):4976–4987. doi: 10.1093/nar/gkt182 23558749 10.1093/nar/gkt182 PMC3643595

[12] Zhou T, Ding JW, Wang XA. Long noncoding RNAs and atherosclerosis. Atherosclerosis. 2016;248:51–61. doi: 10.1016/j.atherosclerosis.2016.02.025 26987066 10.1016/j.atherosclerosis.2016.02.025

[13] Krell J, Frampton AE, Mirnezami R, Harding V, De Giorgio A, Roca Alonso L, et al Growth arrest-specific transcript 5 associated snoRNA levels are related to p53 expression and DNA damage in colorectal cancer. PLoS One. 2014 6 13;9(6):e98561 doi: 10.1371/journal.pone.0098561 eCollection 2014. 24926850 10.1371/journal.pone.0098561 PMC4057237

[14] Amaral PP, Clark MB, Gascoigne DK, Dinger ME, Mattick JS. lncRNAdb: a reference database for long noncoding RNAs. Nucleic Acids Res. 2011;39. 10.1093/nar/gkq1138 PMC3013714 21112873

[15] Williams GT, Mourtada-Maarabouni M, Farzaneh F. A critical role for non-coding RNA GAS5 in growth arrest and rapamycin inhibition in human T-lymphocytes. Biochem. Soc. Trans. 2011;39 (2):482–486. doi: 10.1042/BST0390482 21428924 10.1042/BST0390482

[16] Li L, Li X, The E, Wang LJ, Yuan TY, Wang SY, et al Low expression of lncRNA-GAS5 is implicated in human primary varicose great saphenous veins. PLoS One.2015;10: e0120550 doi: 10.1371/journal.pone.0120550 25806802 10.1371/journal.pone.0120550 PMC4373870

[17] De Jong OG, Van Balkom BW, Schiffelers RM, Bouten CV, Verhaar MC. Extracellular vesicles: Potential roles in regenerative medicine. Frontiers in Immunology. 2014;5:608 doi: 10.3389/fimmu.2014.00608 25520717 10.3389/fimmu.2014.00608 PMC4253973

[18] Ahadi A, Khoury S, Losseva M, Tran N. A comparative analysis of lncRNAs in prostate cancer exosomes and their parental cell lines. Genomics. 2016;9:7–9. 10.1016/j.gdata.2016.05.010 PMC4909828 27330995

[19] Chen L, Yao H, Hui JY, Ding SH, Fan YL, Pan YH, et al Global transcriptomic study of atherosclerosis development in rats. Gene. 2016;592(1):43–48. doi: 10.1016/j.gene.2016.07.023 27425867 10.1016/j.gene.2016.07.023

[20] Gezer U, Özgür E, Cetinkaya M, Isin M, Dalay N. Long non-coding RNAs with low expression levels in cells are enriched in secreted exosomes. Cell Biol Int. 2014;9;38(9):1076–9. doi: 10.1002/cbin.10301 Epub 2014 May 13. 24798520 10.1002/cbin.10301

[21] Cardilo-Reis L, Gruber S, Schreier SM, Drechsler M, Papac-Milicevic N, Weber C, et al Interleukin-13 protects from Interleukin-13 protects from atherosclerosis and modulates plaque composition by skewing the macrophage phenotype. EMBO Molecular Medicine. 2012;4(10):1072–1086. doi: 10.1002/emmm.201201374 23027612 10.1002/emmm.201201374 PMC3491837

[22] Khan R, Spagnoli V, Tardif JC, L’Allier PL. Novel anti-inflammatory therapies for the treatment of atherosclerosis. Atherosclerosis. 2015;240(2):497–509. doi: 10.1016/j.atherosclerosis.2015.04.783 25917947 10.1016/j.atherosclerosis.2015.04.783

[23] Saha P, Modarai B, Humphries J, Mattock K, Waltham M, Burnand KG, et al The monocyte/macrophage as a therapeutic target in atherosclerosis. Current Opinion on Pharmacology. 2009; 9(2):109–118. 10.1016/j.coph.2008.12.017 19230773

[24] Mehta A, Yang B, Khan S, Hendricks JB, Stephen C, Mehta JL. Oxidized low-density lipoproteins facilitate leukocyte adhesion to aortic intima without affecting endothelium-dependent relaxation. Role of P-selectin. Arteriosclerosis, Thrombosis, and Vascular Biology. 1995;15(11):2076–2083. 7583592 10.1161/01.atv.15.11.2076

[25] Ayari H. Respective roles of cortisol, aldosterone and angiotensin II during pathophysiology of atherosclerosis. Ann. Biol. Clin. Paris. 2013;71:381–388. doi: 10.1684/abc.2013.0868 23906564 10.1684/abc.2013.0868

[26] Sun X, Belkin N, Feinberg MW. Endothelial microRNAs and atherosclerosis. Curr. Atheroscler. Rep. 2013;15:372 doi: 10.1007/s11883-013-0372-2 24158362 10.1007/s11883-013-0372-2 PMC3859467

[27] Bianchessi V, Badi I, Bertolotti M, Nigro P, D’Alessander Y, Capogrossi MC, et al The mitochondrial lncRNA ASncmtRNA-2 is induced in aging and replicative senescence in endothelial cells, J. Mol. Cell Cardiol. 2015;81:62–70. doi: 10.1016/j.yjmcc.2015.01.012 25640160 10.1016/j.yjmcc.2015.01.012

